# Confinement-Induced Self-Assembly of Protein Nanofibrils Probed by Microfocus X-ray Scattering

**DOI:** 10.1021/acs.jpcb.4c04386

**Published:** 2025-01-14

**Authors:** Saeed Davoodi, Eirini Ornithopoulou, Calvin J. Gavillet, Anton Davydok, Stephan V. Roth, Christofer Lendel, Fredrik Lundell

**Affiliations:** † Department of Engineering Mechanics, KTH Royal Institute of Technology, 100 44 Stockholm, Sweden; ‡ Wallenberg Wood Science Center, KTH Royal Institute of Technology, 100 44 Stockholm, Sweden; § Department of Chemistry, KTH Royal Institute of Technology, 100 44 Stockholm, Sweden; ∥ Deutsches Elektronen-Synchrotron, D-22607 Hamburg, Germany; ⊥ Institute of Materials Research, Helmholtz-Zentrum Geesthacht, D-22607 Hamburg, Germany; # Department of Fibre and Polymer Technology, KTH Royal Institute of Technology, 100 44 Stockholm, Sweden

## Abstract

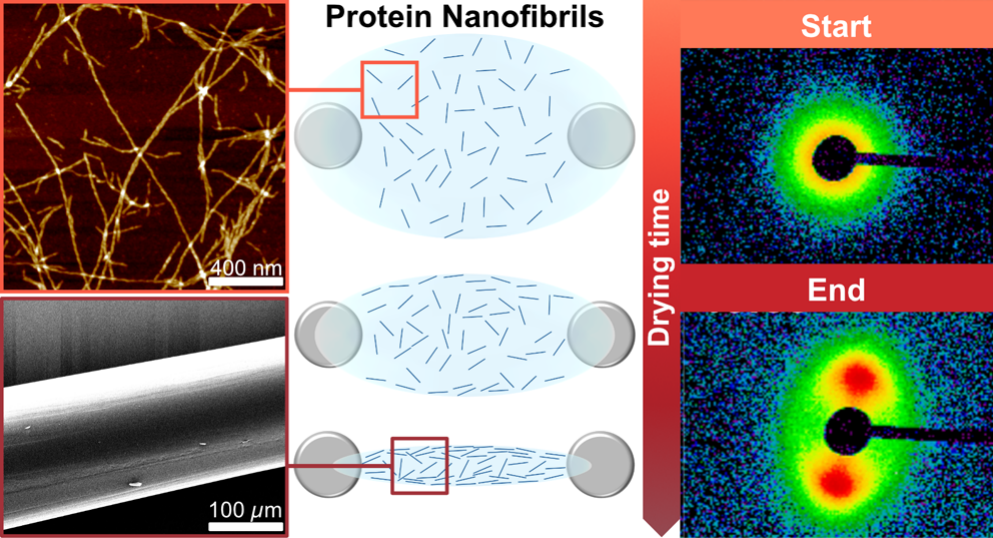

We here explore confinement-induced assembly of whey protein nanofibrils (PNFs) into microscale fibers using microfocused synchrotron X-ray scattering. Solvent evaporation aligns the PNFs into anisotropic fibers, and the process is followed in situ by scattering experiments within a droplet of PNF dispersion. We find an optimal temperature at which the order parameter of the protein fiber is maximized, suggesting that the degree of order results from a balance between the time scales of the forced alignment and the rotational diffusion of the fibrils. Furthermore, the assembly process is shown to depend on the nanoscale morphology and flexibility of the PNFs. Stiff/straight PNFs with long persistence lengths (∼2 μm) align at the air–water interface, with anisotropy decreasing toward the center of the droplet as Marangoni flows increase entanglement toward the center. By contrast, flexible/curved PNFs with shorter persistence lengths (<100 nm) align more uniformly throughout the droplet, likely due to enhanced local entanglements. Straight PNFs pack tightly, forming smaller clusters with short intercluster distances, while curved PNFs form intricate, adaptable networks with larger characteristic distances and more varied structures.

## Introduction

The utilization of proteins to build high-performance materials in nature relies on creating hierarchical structures with well-defined characteristics from the intramolecular conformations (e.g., α-helices, β-strands) up to macroscopic materials with sophisticated properties, such as muscle fibers or spider silk.^1,2^ The ability of proteins to self-assemble into highly ordered protein nanofibrils (PNFs) under certain conditions is associated with amyloid pathologies, including Parkinson’s disease, Alzheimer’s disease, and prion diseases.^3^ However, the amyloid state is a generic, low-energy conformation of proteins,^4^ serving functional or structural purposes related to normal biological behavior, such as melanosome biogenesis^5^ or protein hormone storage.^6,7^ This allows for exploiting this inherent ability of proteins to design and produce functional protein materials, or composite materials intended, e.g., drug delivery applications,^8^ depollution and sustainable materials,^9,10^ solar energy harvesting,^11,12^ tissue-engineering scaffolds,^13^ and biosensors.^14^ Although producing PNFs from protein building blocks can be considered straightforward today, the controlled assembly of the produced nanofibrils into larger, hierarchical structures is challenging.

The fact that many abundant food and plant proteins can form amyloid-like PNFs has highlighted their potential in the development of sustainable materials.^15,16^ Examples of such proteins are those from whey, casein, potatoes, and various legumes.^10,17−19^ Whey protein isolate (WPI) and its main protein component, β-lactoglobulin, is a frequently explored source of PNFs.^20^ The structure of β-lactoglobulin, rich in β-sheets, contributes to the fibrillar morphology and mechanical properties of the PNFs. High β-sheet content promotes stiff, rod-like fibrils with long persistence lengths, likely due to strong hydrogen bonding in aligned backbones. In contrast, at higher concentrations, worm-like fibrils form with less β-sheet content, shorter persistence lengths, and intrinsic curvature, suggesting weaker intermolecular forces. Fibrils are formed at low pH and high temperature, and the fibril morphology can be controlled by changing the concentration of the protein solution.^21,22^ At low concentrations, long, stiff “straight” PNFs are obtained, while higher concentrations yield shorter, flexible, worm-like “curved” PNFs. The stiffness of these two morphological classes was quantified by their persistence length: the straight PNFs have a persistence length of approximately 2 μm, which is about 50 times longer than that of the curved PNFs (ca. 40 nm). This correlation between β-sheet structure and persistence length highlights how secondary structure impacts the flexibility and assembly behavior of PNFs.^21,23^

The nanoscale morphology will affect the structure and properties of materials assembled from the PNFs. For example, we have observed the formation of distinct structural features in solution cast films.^24^ We have also reported substantial differences in the mechanical properties of microfibers assembled from the PNFs by hydrodynamically assisted alignment of the fibrils by means of a double-flow focused microfluidics device.^23,25^ Surprisingly, the curved PNFs were found to form stronger fibers (in the absence of chemical cross-linkers), and it was suggested that the physical entanglement of the more flexible fibrils reinforced the mechanical strength of the spun fiber.

An improved understanding of the assembly mechanisms of PNF-based materials requires experimental characterization at different length scales. One classical method to reveal the atomic distances in the cross-β structure of amyloid-like PNFs is fiber X-ray diffraction experiments.^26^ The experimental approach relies on the formation of anisotropic fibril orientation, for example, by letting a droplet of PNF dispersion dry and form a fiber between two horizontal anchor points.^27,28^ Similar approaches, also known as liquid bridge-induced assembly,^29^ have turned out to be attractive for the creation of nanowire arrays, from, e.g., polyvinyl formal,^30^ DNA,^31^ or spider silk.^32^ At a larger length scale, in situ microfocus small-angle X-ray scattering (SAXS) experiments have turned out to be extremely valuable for examination of the alignment process of fibrillar structures under flow.^23,33−35^ Our studies on PNFs can benefit from the theories developed for liquid crystals, particularly in understanding the alignment and phase transitions of PNFs in solution.^36^ Onsager theory,^37^ based on excluded volume and originally developed for rigid rods, explains the anisotropic interactions between PNFs and the conditions under which they align or aggregate into ordered structures. These concepts are integral to understanding the observed phase behavior of the PNFs under confinement and increasing concentration, especially as the system moves closer to equilibrium during solvent evaporation.

In this work, we explore the alignment and packing behavior of two types of WPI PNFs during solvent evaporation in a droplet, using a setup that combines fiber diffraction sample preparation with in situ microfocusing synchrotron X-ray scattering. We examine the confinement-induced assembly of PNFs with distinct persistence lengths, which directly influence their flexibility and structural behavior. The first type, termed straight PNFs, has a rigid, linear structure, while the second type, termed curved PNFs, adopts more variable conformations. This distinction between stiff/straight PNFs and flexible/curved PNFs provides insight into how persistence length governs the fibril alignment and packing dynamics within confined environments.

SAXS and wide-angle X-ray scattering (WAXS) experiments combined with temperature control allow us to determine how different evaporation rates affect the assembly mechanism, fibril alignment, and packing behavior of the protein fiber hierarchical structure. As the droplet evaporates and the shape of the droplet becomes elongated, the protein concentration is expected to increase at the droplet surface. The concentration gradient and rate of change affect the conformation dynamics of the fibrils at the inner droplet. This process is expected to force the fibril alignment in the outer region of the droplet initially. It is important to note that, unlike liquid crystal studies under equilibrium, our system involves an evaporating droplet, resulting in dynamic conditions that impact the organization of PNFs.

## Materials and Methods

### Preparation of WPI Protein Nanofibrils

WPI (Lacprodan DI-9224) was kindly provided by Arla Food Ingredients. The protein powder was dissolved in pH 2.0 HCl solution at a high concentration (>100 g L^–1^) and dialyzed against 10 mM HCl using a 6–8 kDa cutoff membrane overnight. Then, the protein concentration was adjusted (to 40 or 93 g L^–1^) by dilution with HCl solution, and the dispersion was incubated at 90 °C for 3 days to allow for fibril formation. The incubation at a low concentration (40 g L^–1^) leads to straight, long fibrils, whereas the high concentration incubation (93 g L^–1^) results in short, flexible, curved fibrils. The WPI nanofibril dispersion was dialyzed using a membrane with 100 kDa cutoff for 4 days with frequent changes of dialysis solution (10 mM HCl, pH 2.0). To compensate for the concentration differences, the sample with straight fibrils was concentrated ×2 by centrifugal membrane filtration using a 100 kDa cutoff filter.

### Electron Microscopy Characterization

For SEM imaging, the dried fiber was stabilized on carbon tape and sputtered with a Pt/Pd target to approximately a 7 nm coating thickness. The images were acquired using a Hitachi 4800 SEM instrument, operating at 3.0 kV.

### Horizontally Anchored Hanging Droplet Setup

The setup was designed and manufactured at our in-house mechanical workshop facilities. The device was wire cut out of a stainless steel plate and roller burnished. Temperature control was established by an attached flexible resistive foil heater with a heating capacity of 0.016 W mm^–2^ (TLK-H, TC300, both Thorlabs Inc.). The protein droplet was placed between the anchor points using a plastic tip Eppendorf mechanical pipette (2–20 μL), and the volume was always set at 8 μL.

### Synchrotron X-ray Scattering

Transmission X-ray scattering measurements were performed at the nanofocus end-station of P03 at Petra III in Hamburg, Germany.^38^ The wavelength was λ = 0.9839 Å (*E* = 12.6 keV), with a beam size of 2.5 × 1.5 μm^2^ (H × V) and a sample-to-detector distance (SDD) of 206 ± 1 mm. The WAXS patterns were recorded by using a Pilatus 1 M (Dectris AG, Switzerland) pixel detector (172 × 172 μm^2^). For SAXS experiments, the same detector was used but at SDD = 657 ± 1 mm. The experiments were conducted by placing 8 μL of the protein solution between the two anchor points of the metal device (Figure S1). Upon drying into a fiber, the nanofibrils were aligned into a microscale fiber.^27,39^ 50 positions were measured with an integration time of 0.5 s point^–1^. The distribution of X-ray intensity over the sample avoided any beam damage.^40^ The temperature of the attachment points was controlled by a TC300 temperature controller (Thorlabs Inc.). The surroundings were constant at 25 °C, and the humidity was between 30 and 35%.

For SAXS and WAXS measurements, the scattering images were corrected by subtracting the corresponding air background images taken during the experiment to accurately isolate the scattering signal of the sample. To account for the changing geometry of the droplet during evaporation, we determined the droplet surface by identifying the first and last points with strong signals and used this consistent approach throughout the drying process. The anisotropic scattering was extracted by radially integrating and plotting the data in intensity versus the azimuthal angle or the *q*-range to extract either alignment or the corresponding sizes, respectively. Areas on the scattering pattern affected by strong surface scattering were neglected in the analysis. The orientation index *f*_*c*_ of PNFs was determined by analyzing intensity distribution profiles and performing azimuthal integration according to the following equation:^41^
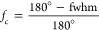
1

where fwhm is the full width at half-maximum of the azimuthal integration. A value of 0 for *f*_c_ suggests a structure that is isotropic, meaning that it lacks orientation or alignment, whereas a value of 1 indicates a structure that is fully aligned.

## Results and Discussion

To understand the phase behavior of PNFs, we examine aspect ratio (), fibril volume fraction, and Onsager critical concentration, which are key parameters relevant to liquid crystalline phase formation.^42,43^ While the system deviates from equilibrium as the droplet evaporates, these parameters offer insight into initial conditions and the system’s proximity to the isotropic-to-anisotropic transition.

Aspect ratio is a key determinant of the phase behavior of rod-like particles. For the straight PNFs, concentrated to an effective 80 g L^–1^, the average length (*L*) is ∼2 μm, and the diameter (*D*) is 4.1 ± 1.1 nm,^23^ resulting in an aspect ratio of ∼488 ± 1. For the curved PNFs at 93 g L^–1^, the length is ∼100 nm, and the diameter is 2.5 ± 0.5 nm,^23^ yielding an aspect ratio of ∼40 ± 8. The high aspect ratio of the straight PNFs supports alignment under confinement, whereas the lower aspect ratio of the curved PNFs favors an isotropic organization. The volume fraction at the start of the experiment affects the interaction potential and alignment. The concentrated straight PNFs (80 g L^–1^) have a comparable volume fraction to the curved PNFs (93 g L^–1^), enhancing the likelihood of interactions and entanglements, which contribute to alignment and phase behavior as evaporation proceeds.

According to Onsager’s theory,^37^ rod-like suspensions transition between phases based on volume fraction. For rods with length *L* and diameter *D*, the isotropic phase exists below , and the nematic phase is above . Between these thresholds lies a coexistence region where isotropic and nematic phases form side by side, often creating nematic microdroplets or tactoids.^36,43^

For the straight PNFs, with an aspect ratio , these thresholds correspond to critical volume fractions of approximately ϕ_I_ ≈ 0.68% (6.8 g L^–1^) for the isotropic phase and ϕ_N_ ≈ 0.92% (9.2 g L^–1^) for the nematic phase. Given the effective starting concentration of 80 g/L, the straight PNFs are well above both thresholds, indicating that the system is well within the nematic phase with locally aligned fibrils already from the outset (Figure S2). For the curved PNFs, with an aspect ratio , the critical concentrations are higher: ϕ_I_ ≈ 8.25% (82.5 g L^–1^) and ϕ_N_ ≈ 11.2% (112 g L^–1^). With a starting concentration of 93 g L^–1^, the curved PNFs lie within the coexistence regime (Figure S2), suggesting the presence of both isotropic and nematic regions, likely forming nematic microdroplets or tactoids due to crowding and local phase separation rather than a uniform transition.

### Droplet Size and Fibril Aggregation

**Figure 1:**
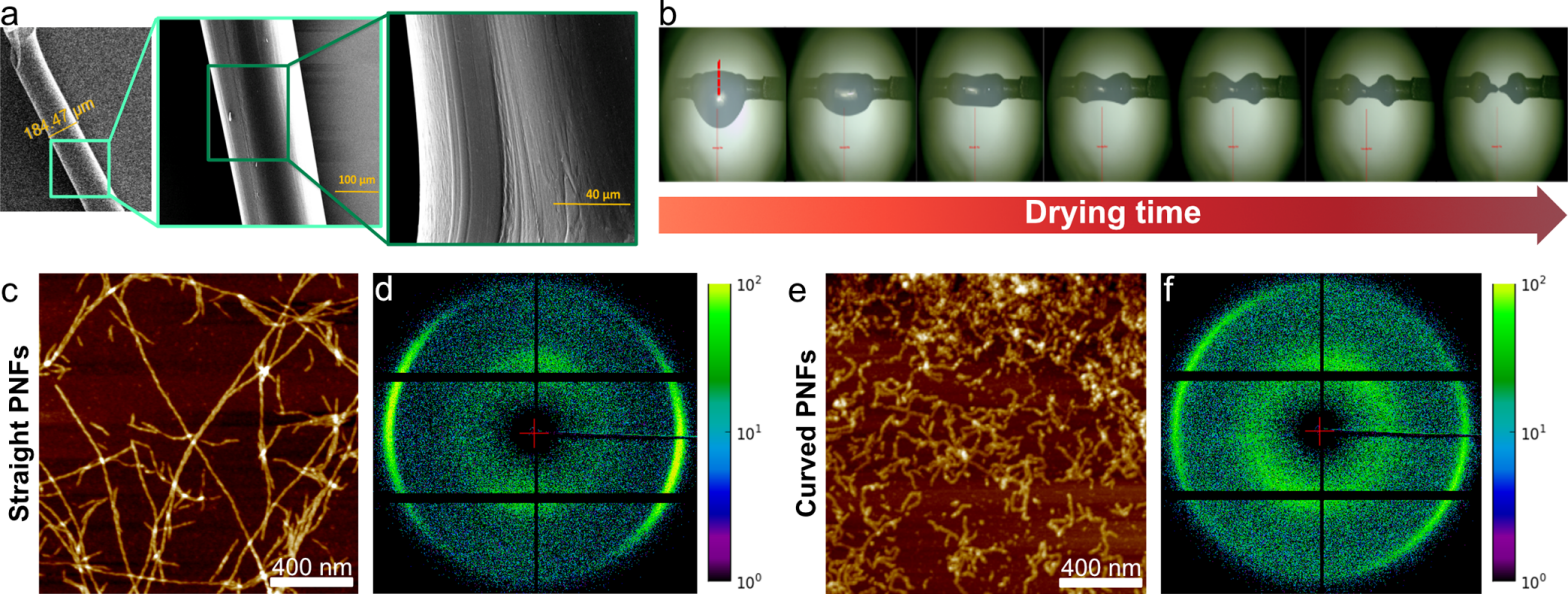
(a) SEM images of a dried fiber. (b) Camera time-lapse images of the evaporation of the solution and the formation of a horizontal protein fiber. AFM images of straight (c) and curved (e) PNFs. WAXS diffraction patterns for dried fibers of straight (d) and curved (f) PNFs.

Preparation of aligned fibrillar structures by letting a droplet of PNF dispersion dry between two horizontal attachment points is an established method for fiber diffraction studies.^27,28^ Glass capillaries sealed with wax or glass rods with rounded edges have previously been used. We successfully prepared fibers using the latter setup. Representative electron micrographs of a formed fiber are shown in Figure 1a. The final diameter is below 1 mm, and the surface is smooth with some anisotropic features.

For the in situ X-ray scattering experiments, we designed a device with metal anchor points to facilitate temperature control and correct beam alignment (Figure S1). The experiments were conducted by placing a droplet (8 μL) of the PNF dispersion between the anchor points in the designed device. Evaporation of the solvent induces the formation of a protein fiber, in which the PNFs become aligned in the horizontal direction (Figure 1b). Typical evaporation times for the droplet were: 27 ± 1 min (25 °C), 20 min (30 °C), 12 min (40 °C), 9 min (50 °C), and 7.4 ± 0.8 min (60 °C) under ambient conditions in the surrounding and a relative humidity of 32 ± 3%. The temperature dependence of the drying time followed an apparent exponential decay (Figure S3). WAXS diffractograms of dried fibers confirm cross-β structure for both classes of PNFs, i.e., straight (Figure 1d) and curved (Figure 1f). The equatorial and meridional reflections translate into the distances 9.02 and 4.67 Å, respectively, for the straight fibrils and 9.49 and 4.67 Å, respectively, for the curved fibrils. These values are in accordance with previously reported distances between individual β-sheets in amyloids.^44^ The shorter distance reports the interstrand separation within the β-sheets, while the longer distance is a measure of the packing of the sheets within a filament.^45^ The slightly longer average intersheet distance observed for the curved PNFs may be related to a less ordered fibril core or different amino acid sequences with consequent changes in the packing of the amino acid side chains.^16^

The small beam size (2.5 × 1.5 μm^2^) allowed us to map the alignment process with a micrometer resolution. As the droplet dried, we scanned it, starting slightly above the air–water interface toward its center (i.e., the vertical midpoint between the two cylindrical metal attachment points) until the bottom (Figure S1c). Measurements were conducted on 50 different points (over a total cross section of 1.2 mm), with each profile acquired over a sampling period of 0.5 s and an average interval of approximately 1 s between consecutive time steps, totaling 75 s for the complete measurement sequence. The final time step in this sequence represents the fully dried droplet, providing a clear end point for the drying process and allowing us to capture the final structural organization of the PNFs. First, we investigated the mass distribution of fibrils within the droplet during the drying process. As temperature increases or time passes (at constant temperature), concentration increases throughout the droplet. Concentration gradients lead to higher concentrations in the outer regions than in the center. The setup might result in localized heating from the anchoring points and might also create temperature gradients across the droplet, which might drive Marangoni convection of the droplet surface from hot to cold areas.^46^ This inward flow would affect the alignment of PNFs, promoting their organization along the radial pathways from the hotter anchor points (low surface tension) to the colder center of the droplet (higher surface tension). These effects were not quantified, but the droplet and its immediate surroundings are probably fairly isothermal, reducing the convection. Furthermore, the viscosity of the droplet increases rapidly as the concentration increases, further reducing convection.

**Figure 2:**
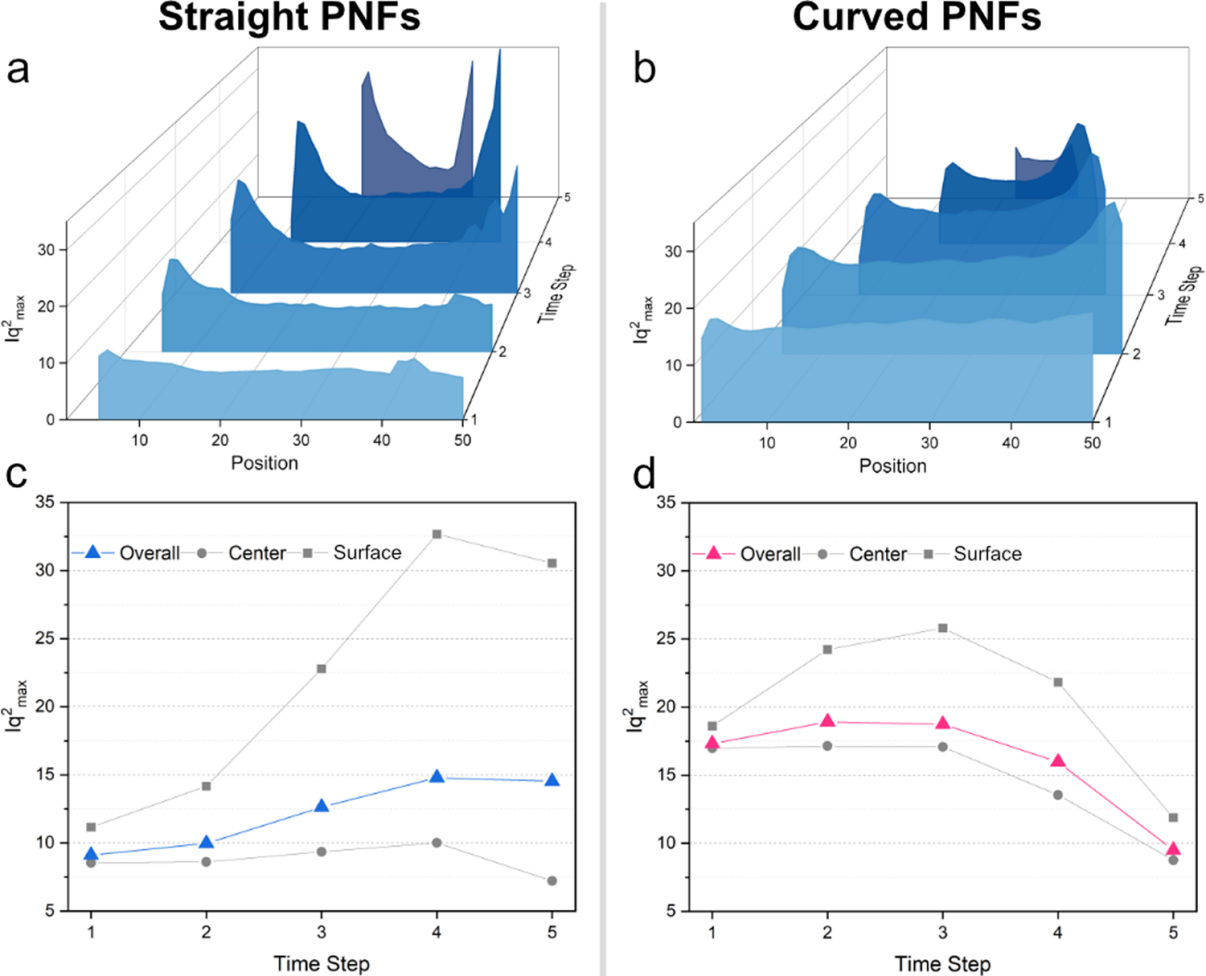
Maximum of the Lorentz-corrected SAXS intensity *Iq*_max_^2^ against scanning position at different time steps during droplet drying at 40 °C for (a) straight and (b) curved PNFs. Averaged *Iq*_max_^2^ over all of the positions, with 10% of the positions closest to the center and 10% of the positions closest to the surfaces of a drying fiber for (c) straight and (d) curved PNFs.

Figure 2 depicts the time dependence of the Lorentz-corrected SAXS curve’s intensity maximum values *Iq*_max_^2^ of drying droplets with straight and curved PNFs at 40 °C (Lorentz-corrected SAXS curves *Iq*_max_^2^ vs scattering vector *q* at 40 °C and different time steps are provided in Figures S4 and S5 for straight and curved PNFs, respectively). We selected this temperature as it demonstrated the highest degree of alignment in a straight PNF droplet, providing insights into the structural differences and alignment dynamics between straight and curved PNF droplets. The nanoscale morphology and flexibility of PNFs significantly affect the structure and properties of the assembled materials. Straight PNFs, with a long persistence length, tend to align at the air–water interface due to their rod-like rigidity, while curved ones with shorter persistence lengths align more uniformly in the droplet, likely due to greater local entanglements. This leads to distinct spatial organization within the droplet and structural anisotropy in the final fiber.

As can be observed in both Figure 2a,b, with evaporation of the water and decreasing droplet size, PNFs (straight, curved) become more prevalent in outer, surface-adjacent regions.^47^Figure 2c,d reveals the distribution trends in more detail: for straight fibrils, *Iq*_max_^2^ increases over time, especially at the surfaces, whereas for curved fibrils, *Iq*_max_^2^ initially rises but decreases over time. The trends in Figure 2 can be explained by the behavior of PNFs at the air–water interface, influenced by Marangoni flow and capillary forces, differential packing, and structural variability. PNFs, especially straight ones, accumulate and align at the interface due to their amphiphilic nature,^16^ rigidity, and high surface tension,^48^ leading to increased *Iq*_max_^2^ values at the surfaces over time (reference studies here). Marangoni flows, caused by surface tension variations from temperature gradients, drive PNFs toward higher surface tension areas at the droplet surfaces, while capillary forces further concentrate them there.^49^ This results in enhanced alignment and packing for straight PNFs, which form tightly aligned clusters (nematic phase) at the surface,^36^ sustaining the rise in *Iq*_max_^2^ (Figure 2c).

Curved PNFs, being more flexible and prone to entanglements, initially accumulate at the surfaces but later show decreased *Iq*_max_^2^ due to less consistent packing, leading to a more isotropic distribution (Figure 2d). This pattern also suggests tactoid-like cluster formation for straight PNFs as they align and concentrate at the surface,^36^ while curved PNFs distribute more uniformly due to their flexibility. The flexibility of curved PNFs allows them to adapt to a horizontal surface more readily than rigid straight PNFs. However, entanglements, combined with a limited time for disentanglement during drying, may restrict their alignment. This entanglement likely contributes to the more isotropic distribution of curved PNFs across the droplet, highlighting the role of structural flexibility and entanglement dynamics in their packing behavior.

**Figure 3:**
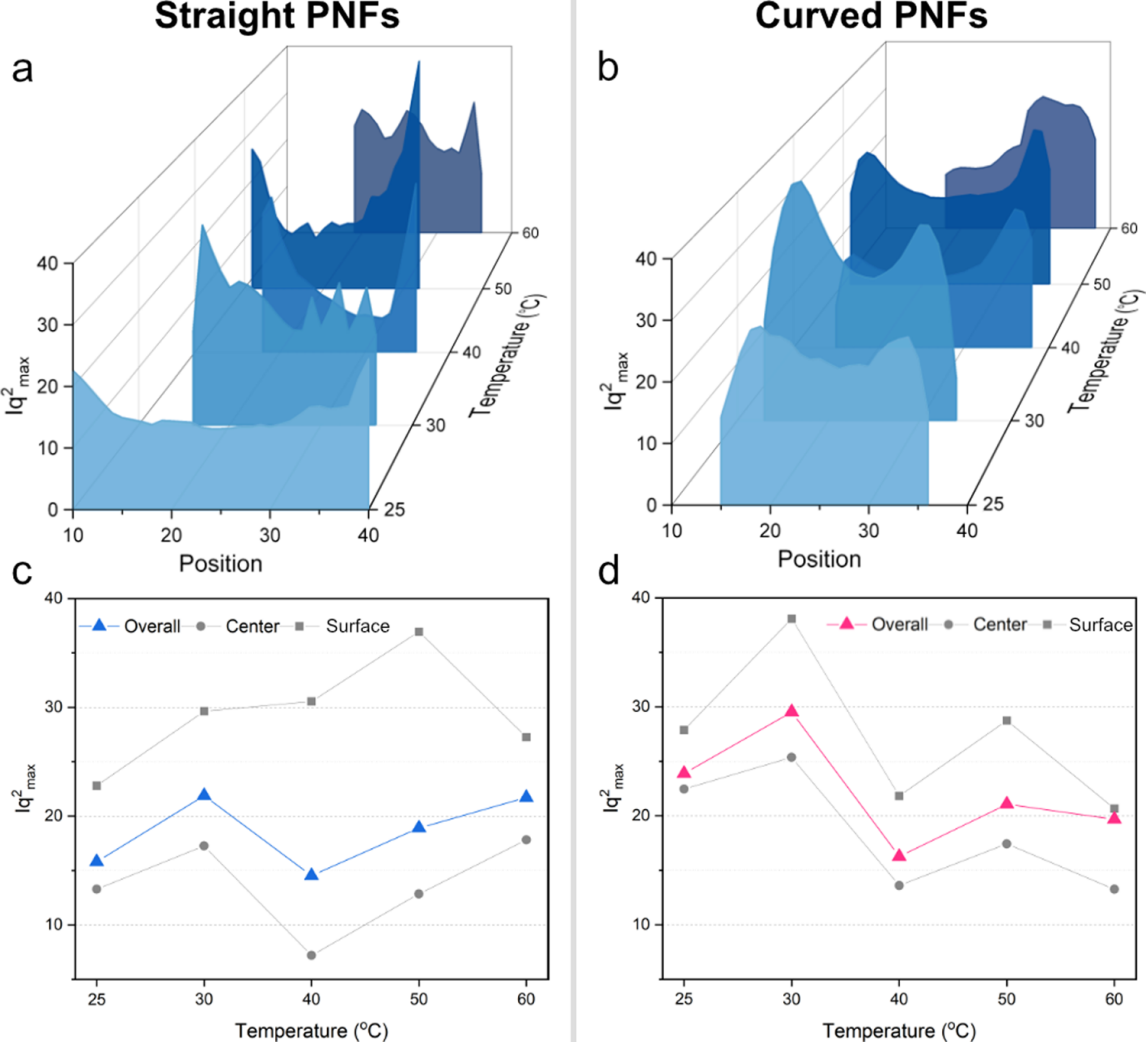
Maximum of the Lorentz-corrected SAXS intensity *Iq*_*max*_^2^ from dried fibers against scanning position for (a) straight and (b) curved PNFs at various temperatures (25, 30, 40, 50, and 60 °C). Averaged *Iq*_max_^2^ over all of the points, with 10% of the positions closest to the center and 10% of the positions closest to the surfaces of the fibers for (c) straight and (d) curved PNFs.

Figure 3 shows the Lorentz-corrected SAXS curve’s intensity maximum values *Iq*_max_^2^ of both straight and curved PNF at different temperatures for final dried fibers. It is clear that all of the dried fibers have almost the same width at the end. Moreover, most structures appear to have formed around the surfaces of droplets based on these plots at all temperatures. At 40 and 50 °C, there is a greater variation between the surface and center of the droplet than at other temperatures for straight and curved PNFs, respectively, suggesting that the concentration gradient might be higher at these temperatures.

### Alignment in Droplets

**Figure 4:**
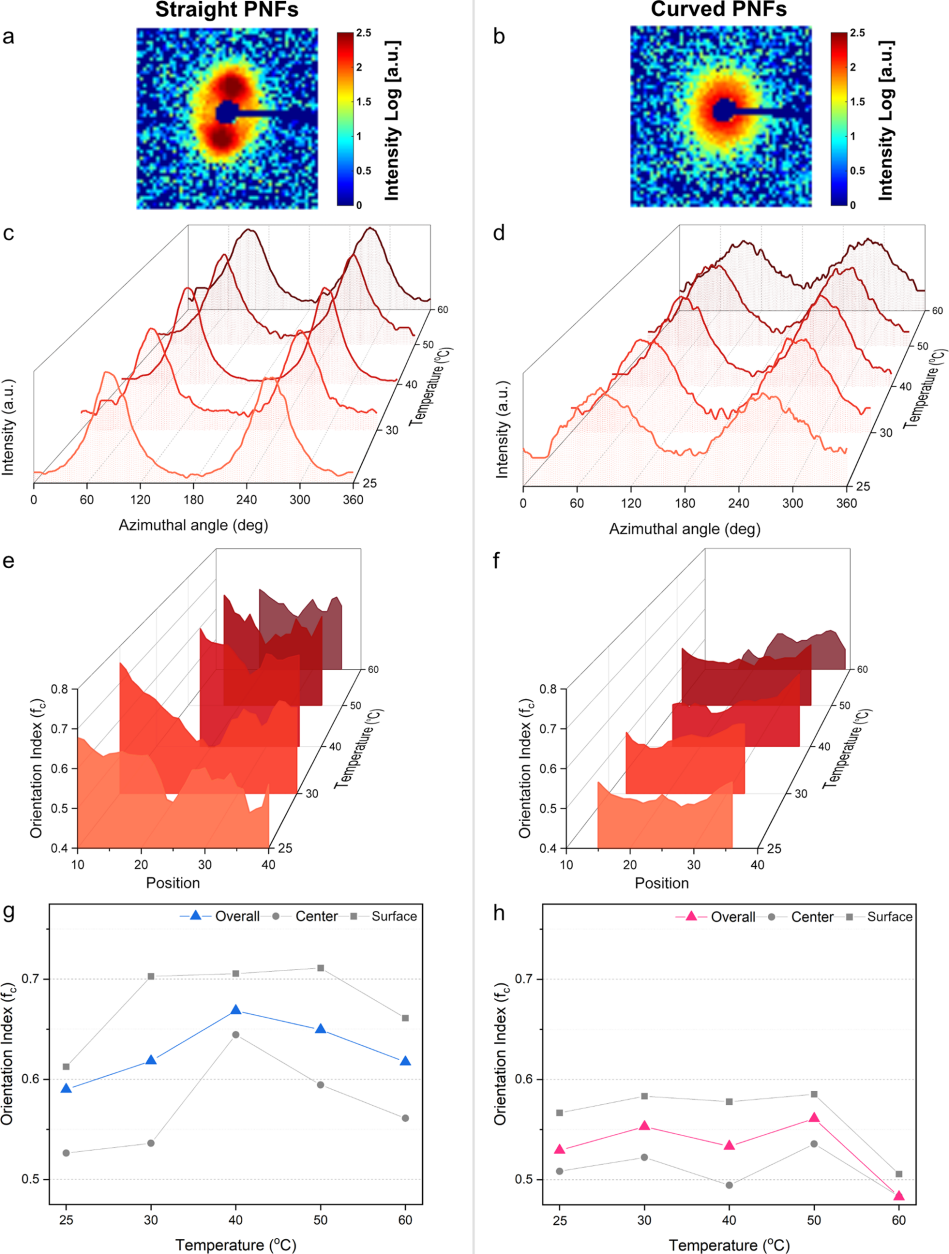
Representative scattering patterns of the dried fibers for (a) straight and (b) curved PNFs. The displayed data is from the experiments at 40 °C. Azimuthal integration of SAXS data of the dried fibers formed at various temperatures (25, 30, 40, 50, and 60 °C) shown for (c) straight and (d) curved PNFs. Orientation index (*f*_c_) plotted as a function of spatial position *z* at different temperatures for (e) straight and (f) curved PNFs. Averaged *f*_c_ over all of the points, with 10% of the positions closest to the center and 10% of the positions closest to the surfaces of dried fibers at various temperatures for (g) straight and (h) curved PNFs.

The starting concentration for straight PNFs is above the critical nematic concentration (*C*_N_), suggesting an initial tendency toward local alignment. For curved PNFs, the concentration is close to *C*_N_, leading to the coexistence of isotropic and nematic regions at the start. This partial alignment is subsequently enhanced by solvent evaporation (and possibly convection flows), which drives further ordering near the droplet surface.^49^ The fiber formation was investigated at different temperatures ranging from 25 to 60 °C. Typical SAXS diffractograms for dried fibers are displayed in Figure 4a,b. Azimuthal integrated SAXS data (Figure 4c,d) of the dried fiber was fitted with two Gaussian curves to determine the full width at half-maximum (fwhm) and then orientation index f_*c*_, which is here used as a measure of alignment of fibrils inside the fiber (see the Methods section for details). Figure 4e,f displays the orientation index values as a function of the temperature for straight and curved PNFs, respectively. The orientation index is clearly lower (i.e., higher fwhm values) for the curved fibrils at all investigated temperatures, which is in agreement with previous studies of the two classes of PNFs under elongational flow.^23^ Our hypothesis was that straight PNFs could better occupy cluster spaces and easily pack close together since they adopt similar orientations in every layer. As a result, alignment between them would be enhanced compared with curved PNFs.

Our observations indicate that the alignment of PNFs is strongly influenced by their persistence length. Straight PNFs, due to their long persistence length, experience alignment primarily at the droplet interface, while curved PNFs display a more uniform alignment across the droplet due to enhanced local entanglements that maintain structural integrity throughout the droplet volume. Interestingly, there seems to be a maximum alignment (minimum fwhm) for both classes of PNFs within the investigated temperature range. For the straight PNFs, the maximum occurs at 40 °C, while the highest alignment for the curved PNFs is found at 50 °C. Both lower and higher temperatures reduce the degree of alignment.

These observations indicate that competing mechanisms are at work. At least three different temperature-dependent processes could be considered: (i) the increased confinement due to solvent evaporation, (ii) the translational diffusion of the PNFs on the length scale of 1 mm, and (iii) the rotational motion of the PNFs. Temperature gradients could also induce bounce or capillary-driven (Marangoni) convection. The evaporation time decreases with increasing temperature, and in our experimental setup, it ranges from a few minutes (at the highest temperature) to ca. 30 min at room temperature (25 °C). The translational diffusion of nanoscale fibrils is significantly slower than their rotational diffusion.^50^ Hence, the translational motions within the droplet will be dominated by the forced motions originating from the shrinking air–water interface. Interestingly, the main time scale for rotational diffusion of β-lactoglobulin PNFs (straight morphology) has been estimated to be ∼10^3^ s (17 min) by stopped-flow birefringence experiments at 20 °C.^51^ This is indeed in the same time regime as the drying time and also shorter than the expected drying time at 25 °C. The time scales of the rotational diffusion are inversely proportional to the temperature,^51^ so it becomes shorter at higher temperatures. However, the exponential decay of the drying time is faster than the change in rotational diffusion, and there is likely a crossover temperature in the investigated temperature regime where the drying time and the main time scale for rotation are approximately equal. The final alignment of the PNFs could then be a temperature-dependent balance between these processes. The time scale for rotational diffusion is also strongly dependent on the fibril length distribution, with short fibrils having faster rotational motions. Hence, the short, curved PNFs are expected to have a higher crossover temperature than the longer straight PNFs. This is in agreement with a higher temperature for the maximum alignment.

At temperatures lower than the optimal range, the rotational diffusion of PNFs is faster than the evaporation rate. This allows the PNFs to undergo more random reorientation as they settle, leading to lower overall alignment as the fibrils lose their organized structure during packing. In contrast, at higher temperatures, the faster evaporation rate limits the time available for fibrils to rotate and align with the shrinking boundary, resulting in entangled, kinetically trapped states that reduce the level of alignment. Thus, optimal alignment occurs at an intermediate temperature, where the drying rate and rotational diffusion are balanced.

As expected, the drying temperature does not affect the molecular organization or the cross-β structure of the fibrils. The amyloid-like structure is stable across room temperature to 60 °C for both the straight and the curved PNFs (Figure S6). The third peak at *q* = 1.7 nm^–1^ (corresponding to 3.75 Å) is associated with repetitive Cα distances in the polypeptide chain and has previously been observed in other amyloid fibrils.^45,52^

**Figure 5:**
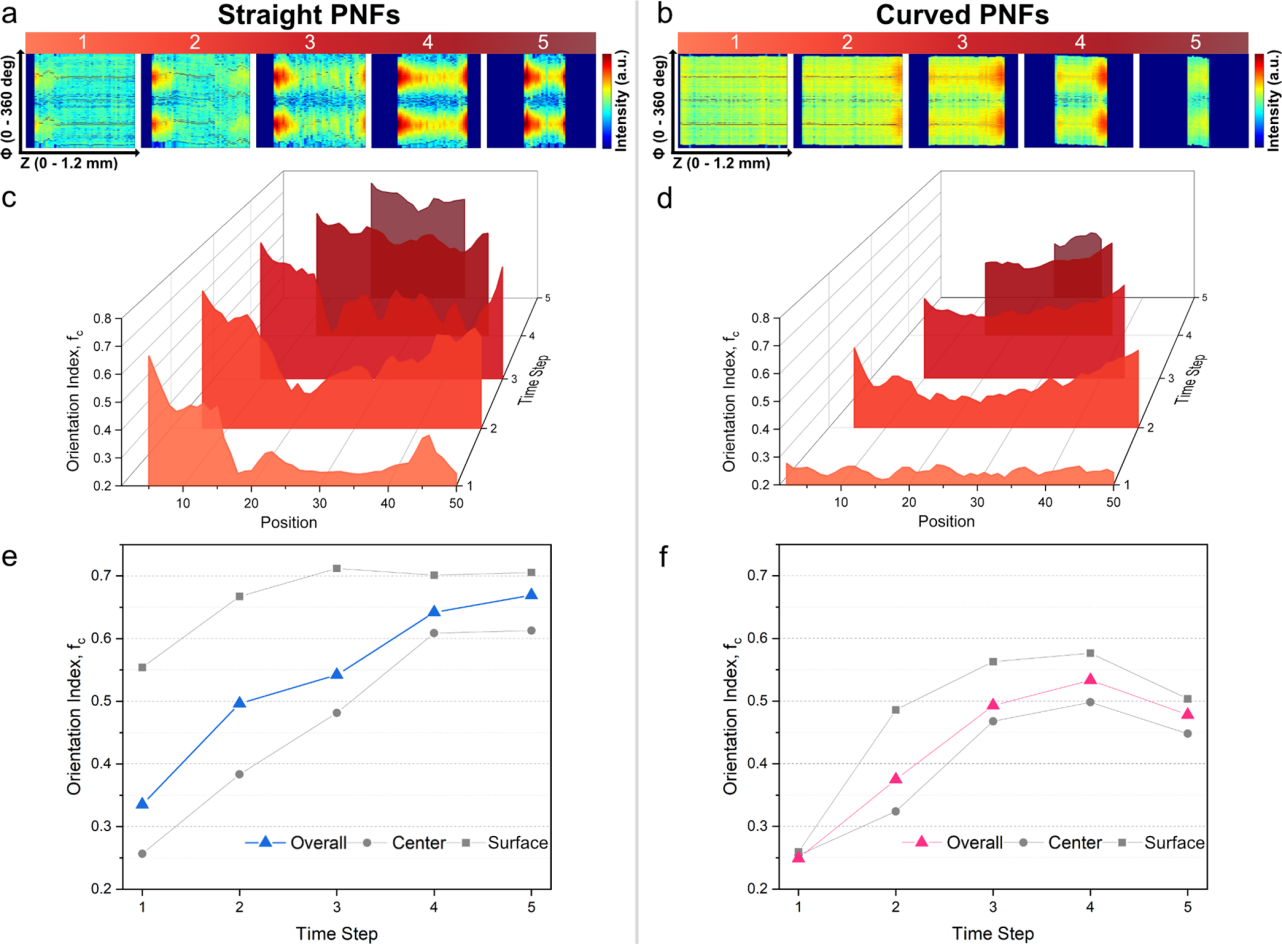
Two-dimensional plots showing the Azimuthal integration (vertical-axis) as a function of position *z* (horizontal-axis) at different time steps during droplet drying and fiber formation at 40 °C for (a) straight and (b) curved PNFs. Orientation index (*f*_c_) plotted as a function of spatial position *z* at the same time points, as shown in (a) and (b) images (same time steps) at 40 °C for (c) straight and (d) curved PNFs. Averaged *f*_*c*_ over all of the points, with 10% of the positions closest to the center and 10% of the positions closest to the surfaces of dried fibers at 40 °C for (e) straight and (f) curved PNFs.

Following the SAXS pattern of the different positions during the evaporation-alignment process reveals the development of an ordered fiber as the droplet size decreases. In the case of the straight PNFs at 40 °C, the SAXS diffraction pattern reveals that the alignment of fibrils begins at the air–water interface of the droplet (Figure 5a,c). The anisotropy is propagated toward the final geometry of the fiber as a consequence of the anisotropic interface contraction. Hence, the moving boundary of the droplet is forcing the PNFs into a compact and aligned state.

Interestingly, the fiber formation process for the curved PNFs appears to be different. Although the anisotropy increases during the drying process, the spatial differences are small, except for very close to the air–water interface (Figure 5b,d). This suggests a different process where alignment occurs as a consequence of PNF packing in the droplet rather than the anisotropic environment at the air–water interface. As the total protein concentrations of the samples are similar, the differences must originate from the nanostructural features. This finding is of interest in relation to the observed assembly mechanisms under elongation flow, as we hypothesized that curved PNFs experience a higher degree of fibrillar entanglement.^23^ This results in distinct structural characteristics of the spun fibers where curved PNFs give surface patterns with micrometer-sized “graupel-like” features, while fibers from straight PNFs display extended anisotropic surface features.^25^ Hence, the assembly of short, curved fibrils may be controlled by local contacts/packing, while the long, straight PNFs are more sensitive to manipulations by external flow fields (elongational flow or droplet shrinking).

### Structure and Confinement in Droplets

As discussed in previous sections, a transition from isotropic to more ordered structures occurs as we move from the center toward the surfaces of the droplet during evaporation. As the process continues, changes in the arrangement and spacing between PNF aggregates are observed. These aggregates align and form structures reminiscent of tactoids, leading to changes in their size and aspect ratio and a significant decrease in spacing between them.^36^ In our observations, the term “tactoid” is used to describe regions where aligned fibrils form within an otherwise isotropic phase, especially near the droplet surfaces. Tactoids are commonly associated with liquid crystal phases, where their formation is governed by local concentration effects and anisotropic interactions between rod-like molecules. In this study, tactoids are observed as elongated, aligned aggregates in the PNF structures, primarily due to the interplay of alignment forces and excluded volume effects, as fibrils aggregate under confinement. This results in localized alignment regions that progressively grow during solvent evaporation, contributing to the characteristic structural anisotropy in the final fiber.

SAXS diffractograms are representations of Fourier transformed electron density distributions in samples at length scales of *d* = 2π/*q*. In all of the Lorentz-corrected SAXS curves (selected Lorentz-corrected SAXS curves (*Iq*^2^ vs *q*) at the final step (dried fiber) and various temperatures are presented in Figures S7 and S8), two characteristics are observed: (i) a shoulder at low *q* values and a peak at higher *q* values. The shoulder corresponds to the size of the PNF clusters formed through aggregation. Straight PNFs had shoulders located at ∼0.186 nm^–1^ corresponding to *d* = 33.5 nm. These fibrils have a characteristic internal structure or periodicity due to their more rigid, linear conformation. On the other hand, for curved PNFs, the shoulder appeared at ∼0.174 nm^–1^, corresponding to *d* = 36 nm, suggesting that there may be a difference in the internal packing or organization. Considering that straight PNFs adopt a similar orientation in each layer, we hypothesized that they would fit better inside bundles since they can occupy the space more efficiently. This would reduce the distance between them in comparison to curved PNFs.

**Figure 6:**
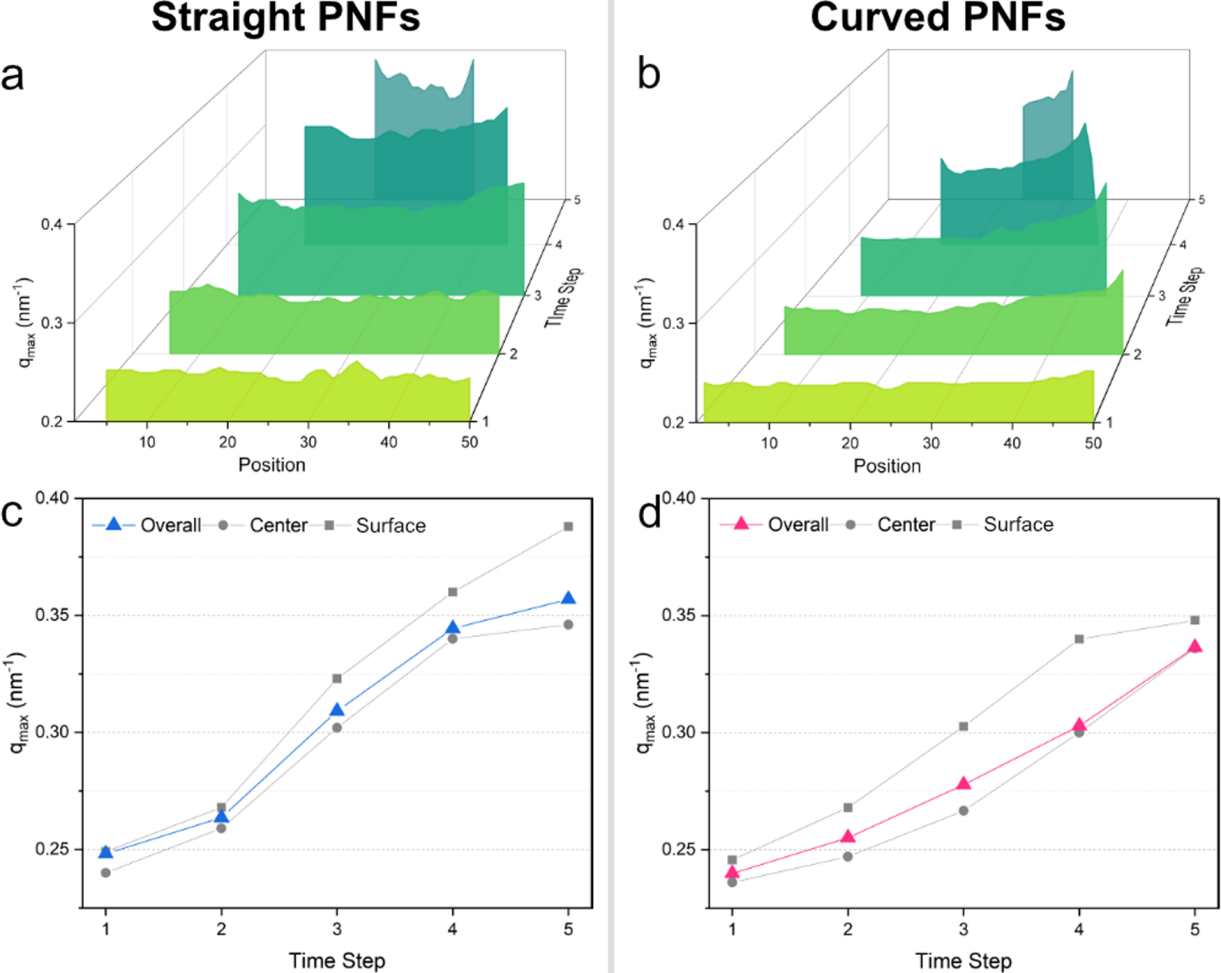
Value *q*_*max*_ at which the Lorentz-corrected SAXS intensity has its maximum vs scanning position at different time steps during droplet drying at 40 °C for (a) straight and (b) curved PNFs. Averaged *q*_*max*_ over all of the positions, with 10% of the positions closest to the center and 10% of the positions closest to the surfaces of drying thread for (c) straight and (d) curved PNFs.

The appearance of peaks at higher *q* values might signal a change in structure such as more compact arrangements or smaller characteristic distances (e.g., distances between fibrils) caused by a higher packing density. Corresponding *q*_max_ values at 40 °C are presented in Figure 6 for both straight and curved PNFs. The movement of the peak toward higher *q* values indicates that repeating structural units, presumably tactoids, are being compressed gradually. As the droplet’s volume decreases due to evaporation, fibrils align and pack more closely, resulting in a denser structure and smaller characteristic distances. This transition from a dispersed state to a compact, aligned state is reminiscent of a liquid crystalline phase. However, unlike an equilibrium liquid crystal, the rapid evaporation process drives the system far from equilibrium, likely leading it to pass briefly through states that resemble liquid crystalline ordering.

Considering the surfaces, *q* values range from 0.25 to 0.39 nm^–1^ and from 0.25 to 0.35 nm^–1^ for straight and curved PNFs, respectively. This indicates that the distance between straight PNFs decreases from 25 to 16 nm, while for curved PNFs, it reduces from 25 to 18 nm. These distance ranges are similar to the calculated interfibril spacing of 22 nm, derived by Yuan et al.^42^ based on Onsager’s theory for equilibrium liquid crystalline phases of amyloid fibrils at a specific volume fraction. In our nonequilibrium system, these distances suggest transient structural ordering that may briefly resemble liquid crystalline phases as fibrils become more closely packed.

As observed, the shift to higher *q* values is more pronounced for straight PNFs than for curved ones. Droplets containing curved fibrils also show larger differences in *q* values between their surfaces and centers compared to those of straight PNFs. This disparity in *q* values reflects a significant variation in characteristic distances within droplets containing curved PNFs, indicating a broader range of structural organization. The broader range in *q*_max_ for curved PNFs primarily arises from concentration gradients as the droplet dries, creating regions with differing PNF densities. Curved PNFs may form an aligned monolayer at the air–water interface early in the drying process, acting as a nucleation site for an ordered phase near the surface. In contrast, within the bulk, entanglements restrict movement, creating kinetically trapped structures with less order. This surface alignment versus bulk entanglement could explain the greater spatial variability seen in curved PNFs, where alignment is stronger near the interface and more isotropic in the interior. Additionally, as the curved PNFs have a larger particle count due to their shorter length, the influence of translational entropy may be enhanced in this sample. This could contribute to the distinct alignment and packing behavior observed, even if translational entropy effects remain secondary in this evaporative, nonequilibrium system. Contrary to this, straight PNFs tend to have more uniform structures, resulting in smaller differences in the characteristic distances across droplets. While thermodynamic packing influences structural differences between straight and curved PNFs, kinetic factors such as flow-induced alignment, rotational diffusion, and convective forces also play a key role. Under nonequilibrium drying, these kinetic effects enhance local alignment and contribute significantly to the distinct structural features observed across the droplet.

**Figure 7:**
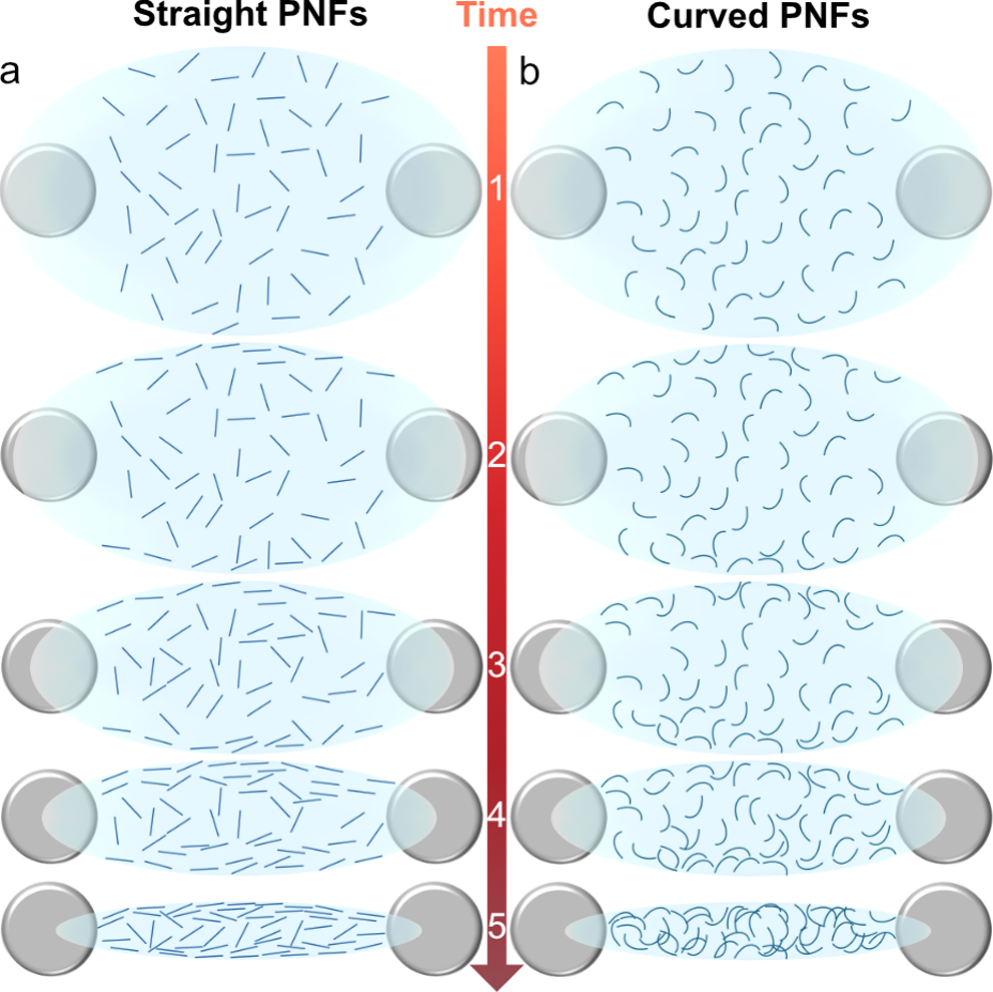
Isotropic + anisotropic phase forms at the surface of the droplet as a result of evaporation. The schematic illustration for (a) straight and (b) curved PNFs.

As the surface area of an evaporating droplet decreases, creating a concentration gradient, the accumulation of PNFs at the surfaces of the droplet leads to a surface concentration gradient that drives flow toward the center.^49^ Consequently, the droplet surfaces and centers display varying degrees of order due to this flow. During the drying process, straight PNFs tend to pack tightly and maintain consistent characteristic distances due to their rigidity and long persistence length (Figure 7a). In contrast, the curved PNFs exhibit increased heterogeneity in aggregate size and form a more variable network structure across the droplet due to their flexibility and adaptability. Often, Marangoni flows increase entanglements as materials move toward the center (from anchor points) because of the curvature of their fibrils. Unlike straight PNFs, curved PNFs form intricate networks, resulting in a larger characteristic distance and a more varied structure across the droplet. These entanglements and network irregularities contribute to regions where fibrils are intertwined in a less ordered pattern (Figure 7b).

Viscosity measurements (Figure S2) indicate that straight and curved PNFs have similar viscosities at low shear rates, while curved PNFs show higher viscosity at high shear rates, likely due to increased entanglements. This higher viscosity under shear could influence flow dynamics during droplet drying, potentially affecting the alignment and packing behavior, particularly in regions with greater shear forces. Understanding the role of entanglements and their variation between curved and straight PNFs is essential for controlling alignment and optimizing the properties of PNF-based materials. While the in situ SAXS data provide detailed insights into the alignment and packing dynamics of PNFs during drying, further investigation would be needed to directly correlate these observations with the mechanical properties of the final dried fibers.

## Conclusions

To conclude, we have explored the evaporation-induced alignment of PNFs into a microscale fiber using a setup similar to what has previously been employed to prepare samples for X-ray fiber diffraction studies of amyloid fibrils. In situ characterization of the fiber formation process reveals that there is a temperature dependence for the degree of alignment in the final fiber, likely because of the balance between solvent evaporation rate and the rotational motions of the PNFs. Our study demonstrates that the confinement-induced assembly of PNFs varies significantly between straight and curved PNFs, with persistence length playing a central role in determining their alignment and packing behaviors. Due to their linear structure and tight packing, straight PNFs form smaller clusters with more consistent interfibrillar distances. The curved PNFs, on the other hand, exhibit greater characteristic distances and enhanced entanglements, resulting in an array of patterns across the droplet due to their inherent flexibility and adaptability. These differences are exacerbated by Marangoni flows, which drive material toward the droplet’s center, enhancing curved PNF networks’ structural complexity. A detailed understanding of these entanglements and how they vary between curved and straight PNFs can inform the design and functionality of materials derived from these processes. By situating our observations within the framework of liquid crystal theory, specifically Onsager’s treatment of rod-like particles and excluded volume effects, we gain insights into the concentration-dependent phase behaviors of straight and curved PNFs. Additionally, the influence of convection and temperature gradients during droplet evaporation underscores the complexity of assembly mechanisms, which include both mass transport and local phase transitions into tactoid-like regions, leading to unique structural variations across the fiber. These findings offer a theoretical and experimental basis for further optimizing assembly conditions to tailor the mechanical properties of PNF-based materials.

## Supporting Information Available

Illustration of experimental setup.; Flow sweep results for straight and curved PNFs.; Evaporation time as a function of temperature; WAXS scattering profiles; Lorentz-corrected SAXS curves (*Iq*_max_^2^ vs *q*) at 40 °C and different time steps for straight PNFs.; Lorentz-corrected SAXS curves (*Iq*_max_^2^ vs *q*) at 40 °C and different time steps for curved PNFs.; Lorentz-corrected SAXS curves (*Iq*_max_^2^ vs *q*) at the final step (dried fiber) and various temperatures (25, 30, 40, 50, and 60 °C) for straight PNFs.; Lorentz-corrected SAXS curves (*Iq*_max_^2^ vs *q*) at the final step (dried fiber) and various temperatures (25, 30, 40, 50, and 60 °C) for curved PNFs (PDF)
