# Supplementary material for: Confinement-Induced Self-Assembly of Protein Nanofibrils Probed by Microfocus X-ray Scattering
Source: J Phys Chem B. 2025 Jan 14;129(3):1070–81. doi: 10.1021/acs.jpcb.4c04386 (PMC11770757; doi:10.1021/acs.jpcb.4c04386)
Supplement: Supplementary file 1 — jp4c04386_si_001.pdf [file jp4c04386_si_001.pdf]

Supporting Information
Confinement-induced Self-assembly of Protein Nanofibrils Probed by Microfocus X-ray Scattering

Saeed Davoodi,[†,‡] Eirini Ornithopoulou,[¶,†] Calvin J. Gavillet,[†,‡,§] Anton Davydok,[‖] Stephan V. Roth,[⊥,§] Christofer Lendel,[*,¶] and Fredrik Lundell[*,†,‡]

†*Department of Engineering Mechanics, KTH Royal Institute of Technology, 100 44 Stockholm, Sweden*

‡*Wallenberg Wood Science Center, KTH Royal Institute of Technology, 100 44 Stockholm, Sweden*

¶*Department of Chemistry, KTH Royal Institute of Technology, 100 44 Stockholm, Sweden*

§*Deutsches Elektronen-Synchrotron, D-22607 Hamburg, Germany*

‖*Institute of Materials Research, Helmholtz-Zentrum Geesthacht, D-22607 Hamburg, Germany*

⊥*Department of Fibre and Polymer Technology, KTH Royal Institute of Technology, 100 44 Stockholm, Sweden*

E-mail: lendel@kth.se; frlu@kth.se

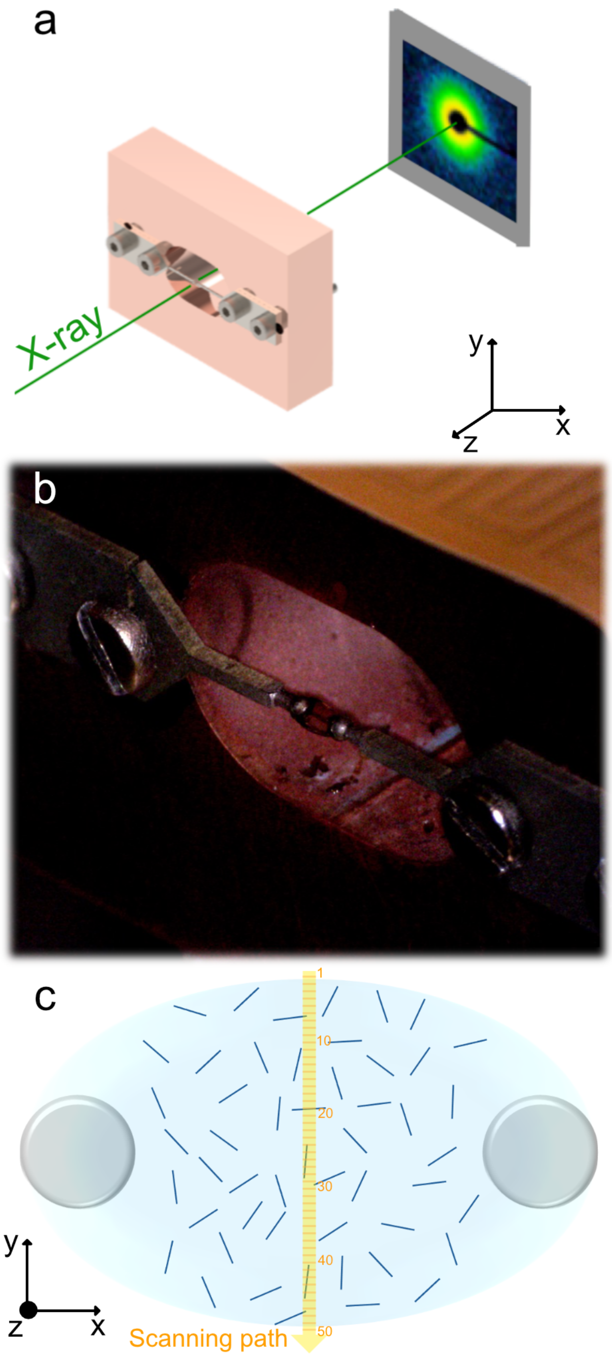

Figure S1: a) Sketch of the experimental setup. b) Photo of the actual device. The copper block with the hole can be observed, along with the aligned stainless steel spherical heads droplet holders in the middle of the device. c) Schematic of scanning direction (The arrow represents the scanning path through the droplet).

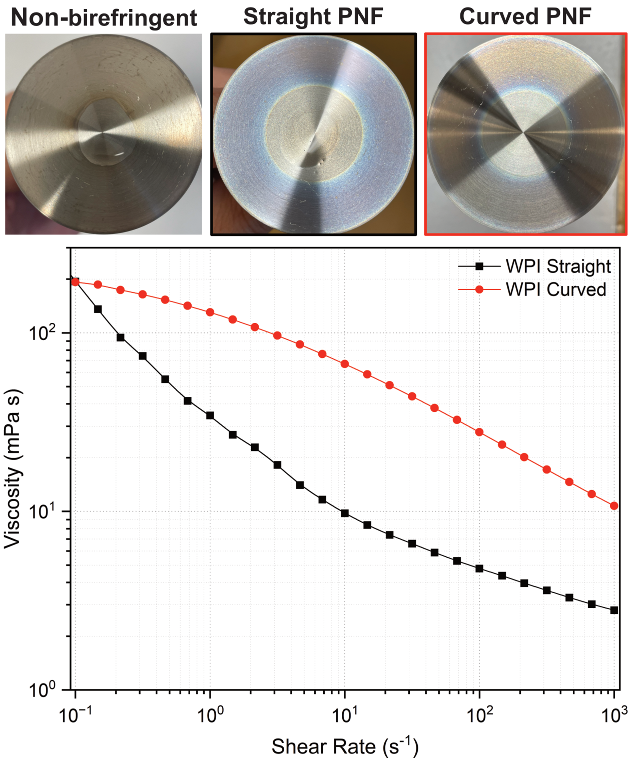

Figure S2: Top: Rheometer geometries post-test: Non-birefringent sample as reference, highly birefringent straight PNFs above C_N, showing pronounced nematic alignment; and slightly birefringent curved PNFs, at a concentration between C_I and C_N. Bottom: flow curves of straight and curved PNFs.

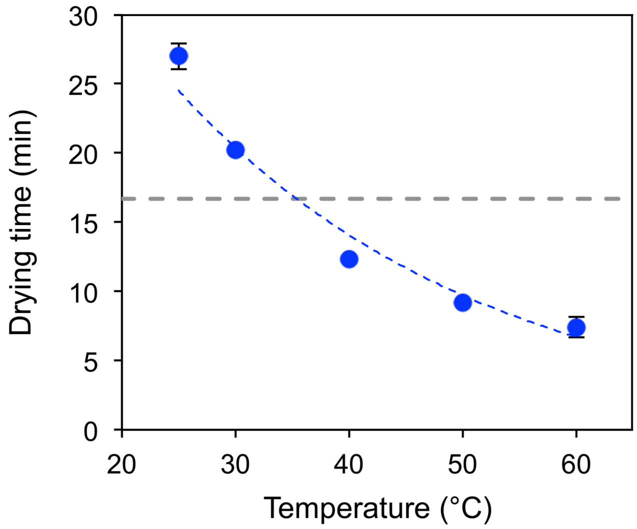

Figure S3: Droplet drying time as function of temperature (blue) and main time regime for rotational motions ($\sim 10^3$ s) at 20 °C of straight PNFs[1] indicated by a grey dashed line.

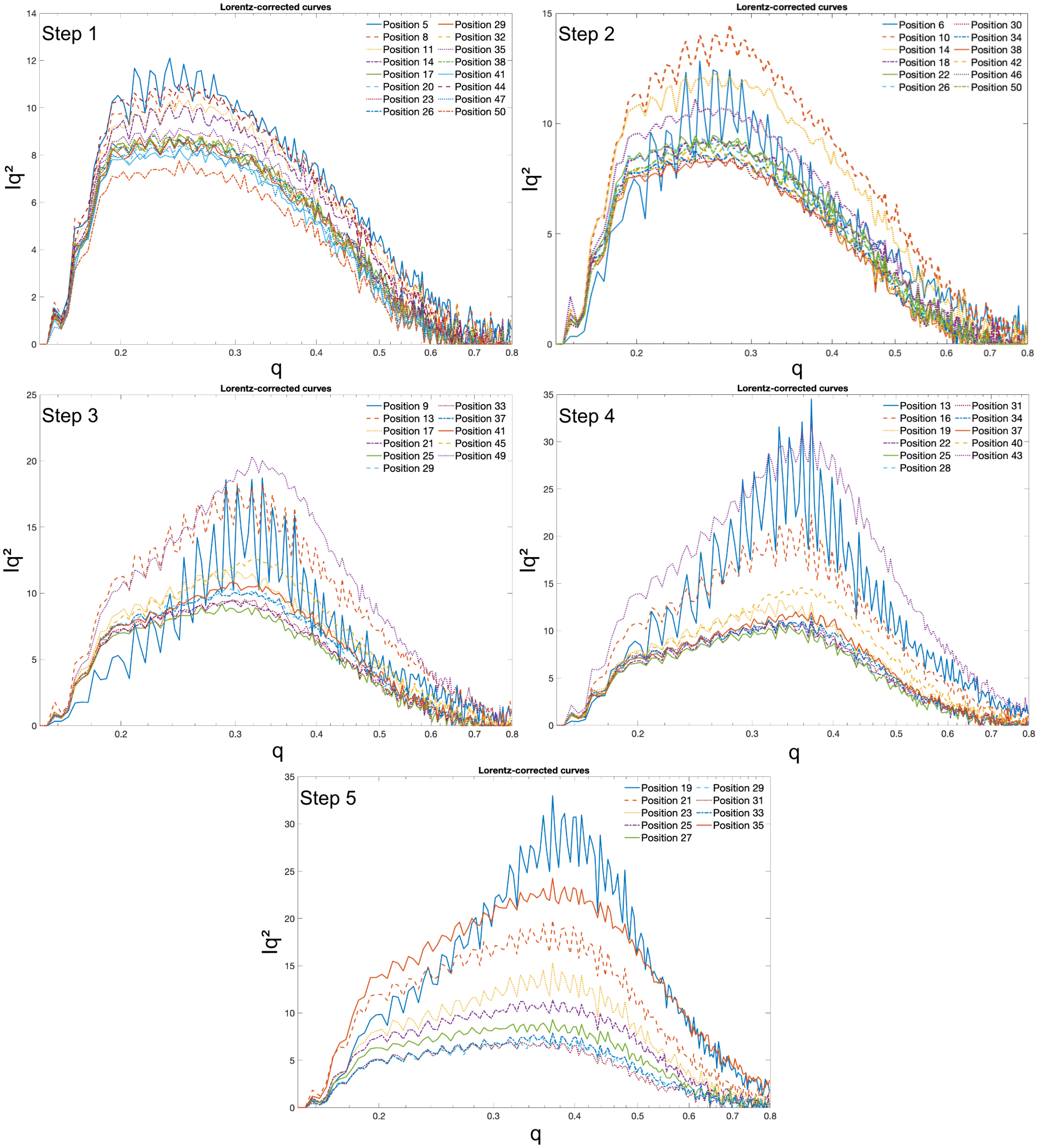

Figure S4: Selected Lorentz-corrected SAXS curves (Iq^2 vs. q) at 40 °C and different time steps for straight PNFs. The shift toward higher Iq^2_{max} values (indicating larger structures) and larger q_{max} values (corresponding to smaller distances) at droplet surfaces can be clearly observed as time passes and it dries.

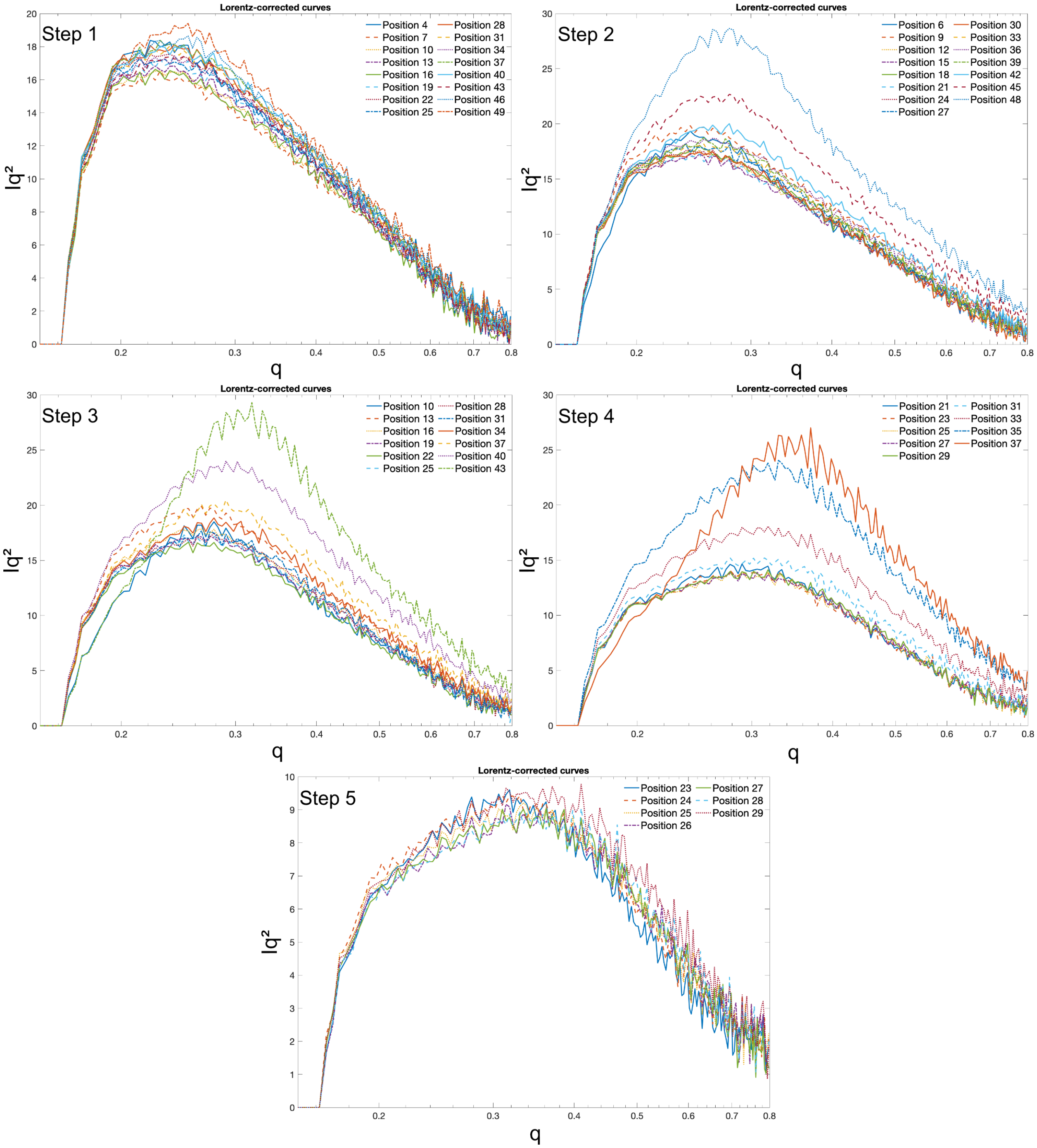

Figure S5: Selected Lorentz-corrected SAXS curves (Iq^2 vs. q) at 40 °C and different time steps for Curved PNFs. The shift toward higher Iq^2_{max} values (indicating larger structures) and larger q_{max} values (corresponding to smaller distances) at droplet surfaces can be clearly observed as time passes and it dries.

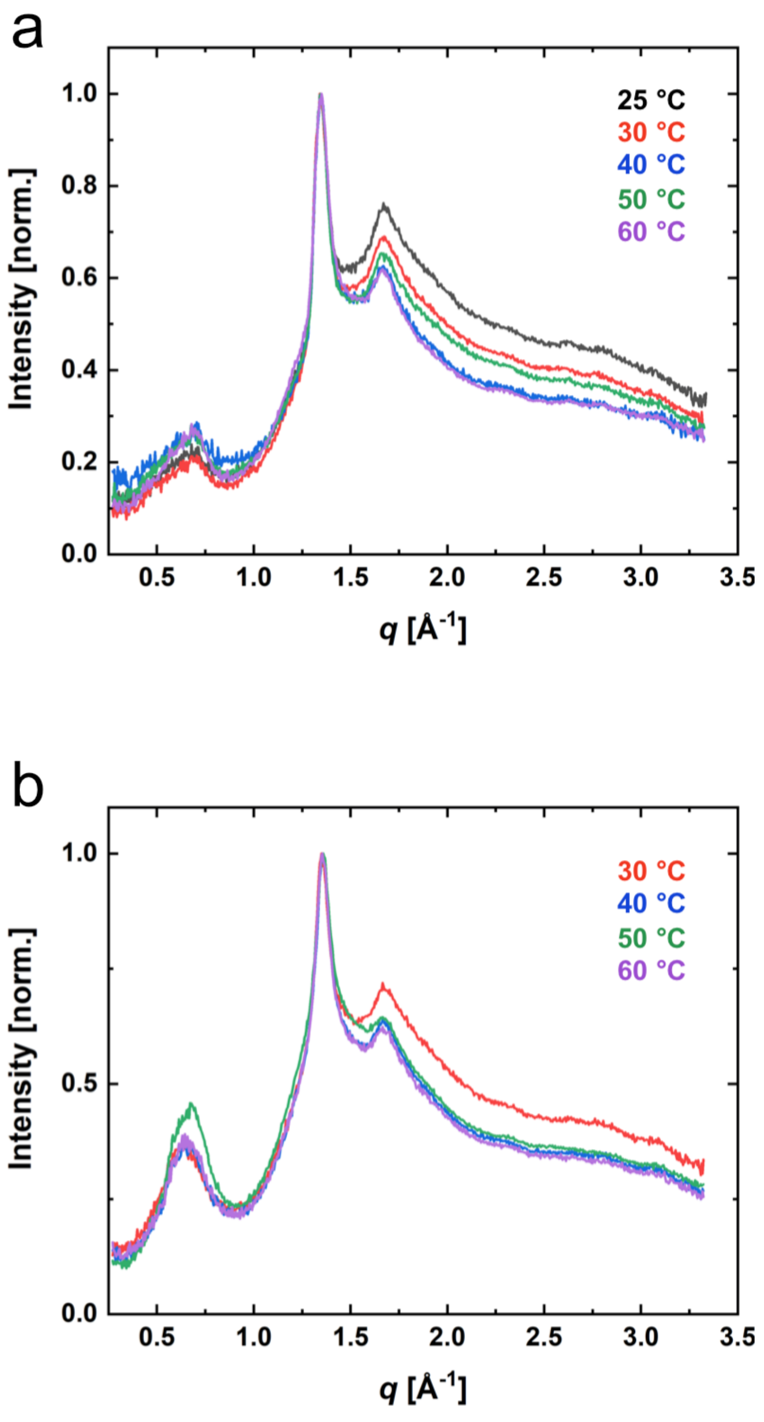

Figure S6: WAXS scattering profiles. Peak normalized intensity over q for different temperatures for (a) straight fibrils and (b) curved fibrils.

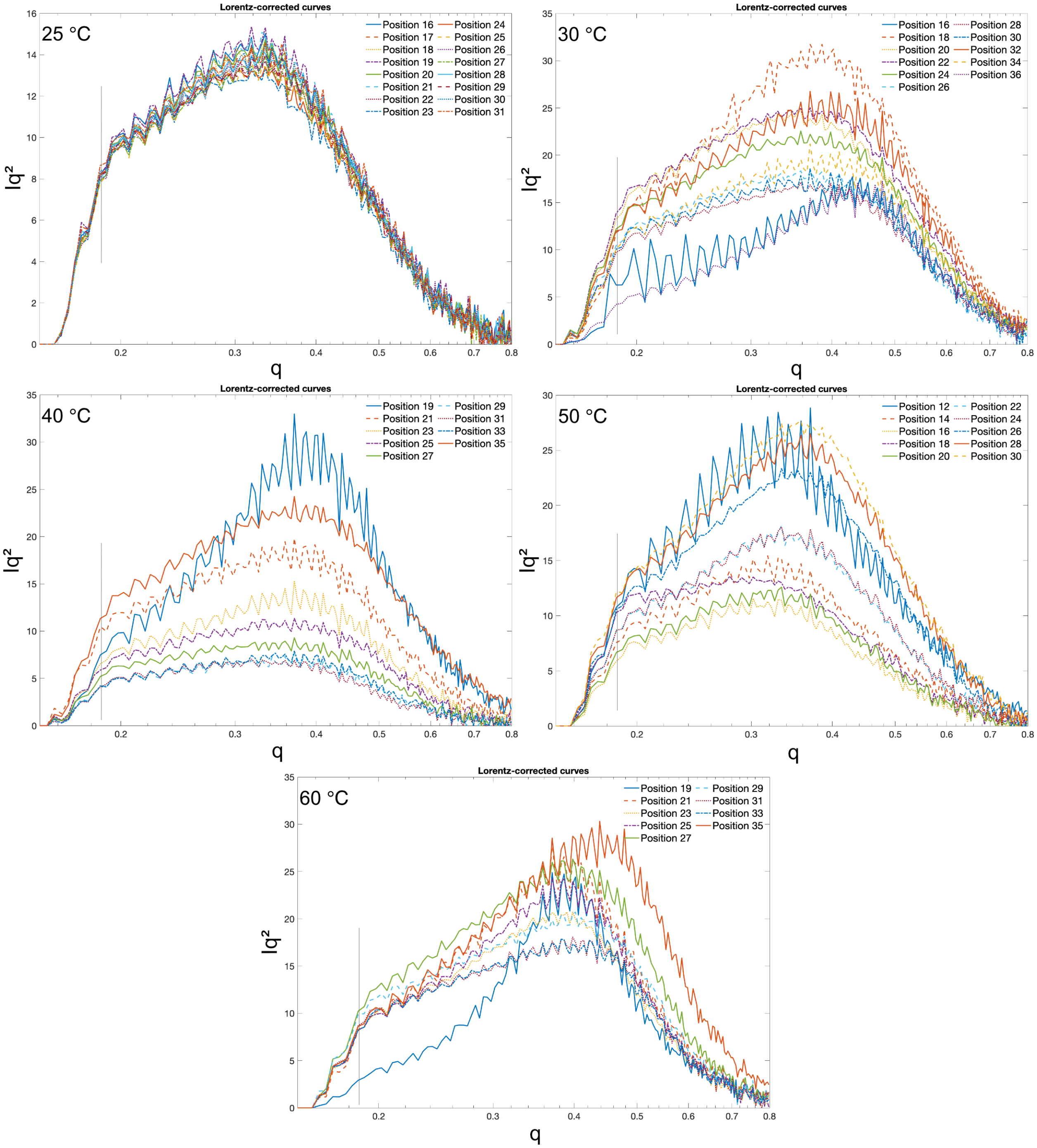

Figure S7: Selected Lorentz-corrected SAXS curves (Iq^2 vs. q) at the final step (dried fiber) and various temperatures for straight PNFs. The shoulder can be seen at 0.186 nm^{-1} (corresponding to $d = 33.5$ nm) across all temperatures.

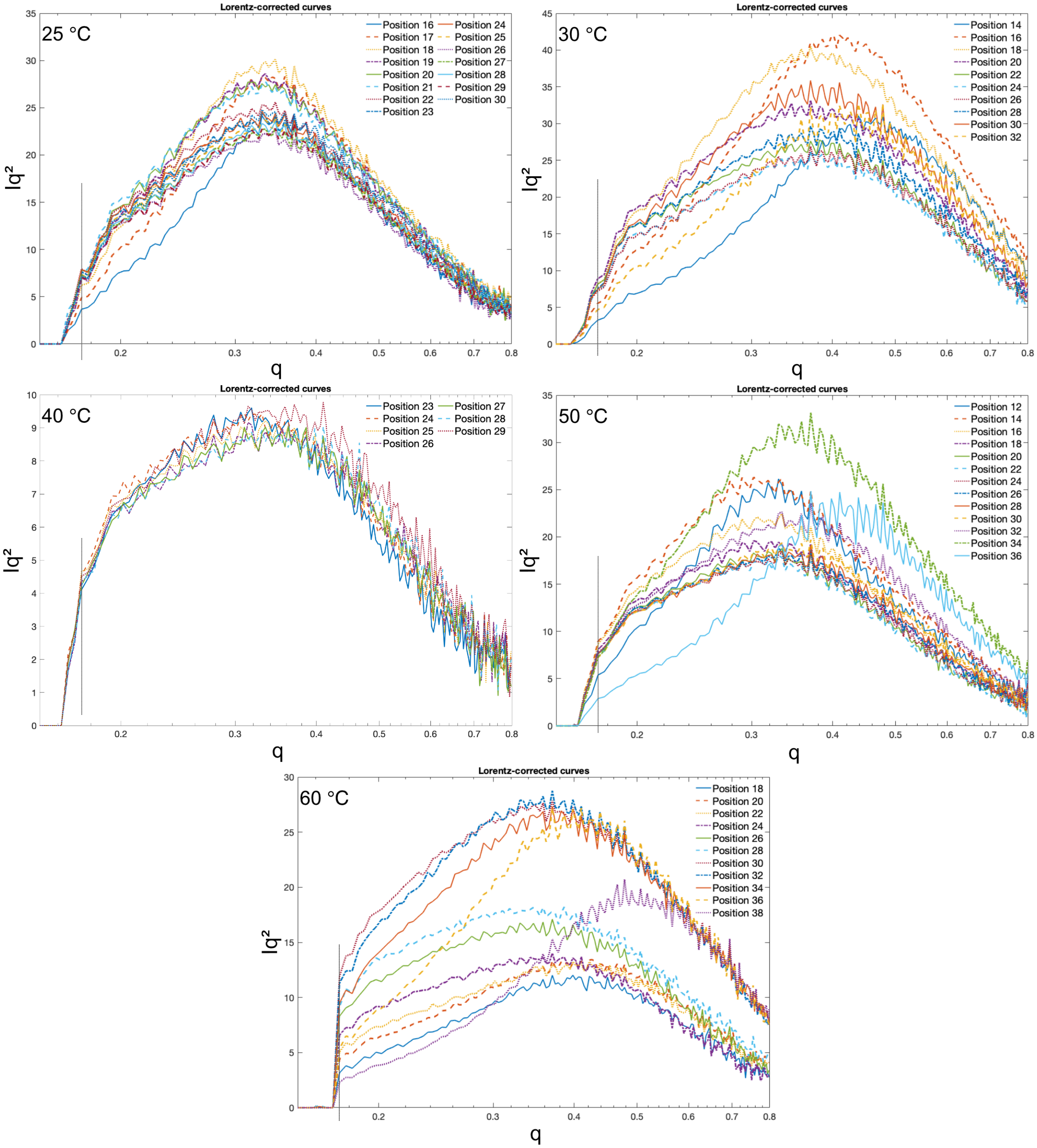

Figure S8: Selected Lorentz-corrected SAXS curves (Iq^2 vs. q) at the final step (dried fiber) and various temperatures for Curved PNFs. The shoulder can be seen at 0.174 nm^{-1} (corresponding to $d = 36$ nm) across all temperatures.

References

(1) Rogers, S. S.; Venema, P.; Sagis, L. M.; Van Der Linden, E.; Donald, A. M. Measuring the length distribution of a fibril system: a flow birefringence technique applied to amyloid fibrils. *Macromolecules* **2005**, *38*, 2948–2958.
